# Analyzing sex imbalance in EGA and dbGaP biological databases: Recommendations for better practices

**DOI:** 10.1016/j.isci.2024.110831

**Published:** 2024-09-23

**Authors:** Victoria Ruiz-Serra, Nataly Buslón, Olivier R. Philippe, Diego Saby, María Morales, Camila Pontes, Alejandro Muñoz Andirkó, Gemma L. Holliday, Aina Jené, Mauricio Moldes, Jordi Rambla, Alfonso Valencia, María José Rementeria, Atia Cortés, Davide Cirillo

**Affiliations:** 1 Life Sciences, Barcelona Supercomputing Center (BSC), 08034 Barcelona, Spain; 2 European Commission, Joint Research Centre (JRC), 2440 Geel, Belgium; 3 Web Science and Social Computing Group Department of Engineering, Universitat Pompeu Fabra (UPF), 08018 Barcelona, Spain; 4 Data Science Unit, LHF Labs S.L, 48020 Bilbao, Spain; 5 Cognitive Biology of Language Group, University of Barcelona, 08007 Barcelona, Spain; 6 Medicines Discovery Catapult, Mereside, Macclesfield SK10 4ZF, UK; 7 Computational Biology and Health Genomics Programme, Centre for Genomic Regulation (CRG), The Barcelona Institute of Science and Technology, 08003 Barcelona, Spain; 8 Institución Catalana de Investigación y Estudios Avanzados (ICREA), 08010 Barcelona, Spain

**Keywords:** Human genetics, Genomics, Artificial intelligence

## Abstract

Precision medicine aims at tailoring treatments to individual patient’s characteristics. In this regard, recognizing the significance of sex and gender becomes indispensable for meeting the distinct healthcare needs of diverse populations. To this end, continuing a trend of improving data quality observed since 2014, the European Genome-phenome Archive (EGA) established a policy in 2018 that mandates data providers to declare the sex of donor samples, aiming to enhance data accuracy and prevent imbalance in sex classification. We analyzed sex classification imbalance in human data from EGA and the U.S. counterpart, the database of genotypes and phenotypes (dbGaP). Our findings show a significant decrease in samples classified as unknown in EGA, potentially promoting better sex reporting during data collection. Based on our findings, we raise awareness of sample imbalance problems and provide a list of recommendations for enhancing biomedical research practices.

## Highlights

• Imbalances in sex metadata can impede the accuracy of precision medicine research
• There have been sex representation imbalances across EGA and dbGaP studies over time
• In recent years, EGA has mandated the reporting of sex data to improve data accuracy
• "Unknown" sex classification in EGA dropped significantly after policy changes

## Graphical abstract

**Figure:**
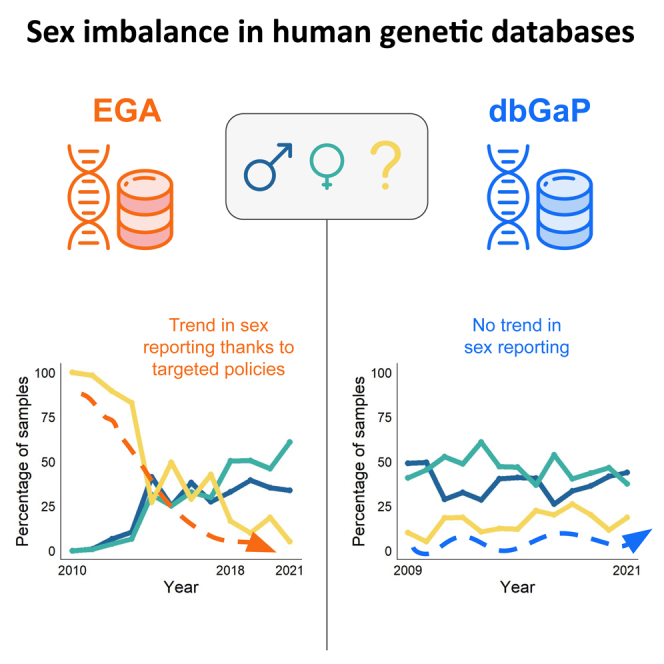

## Introduction

In the area of human data collection, two of the largest databases worldwide are the Database of Genotypes and Phenotypes (dbGaP), hosted at the National Center for Biotechnology Information (NCBI),^1^ and the European Genome-phenome Archive (EGA), hosted at the European Bioinformatics Institute (EMBL-EBI) and the Barcelona Supercomputing Center (BSC).^2^ The Global Alliance for Genomics & Health (GA4GH) defines EGA and dbGaP as “the main public genome archives” globally (www.ga4gh.org). These widely used resources play a crucial role in biomedical research. However, like any other biological data resource, their effectiveness relies on diligent maintenance and efficient operation through solid data management practices.^3^ Indeed, the quality and content of such data have an immense impact on the biomedical insights that can be derived from their analysis. Additionally, if such data is not collected properly, it can pose a significant risk of bias, consequently impacting critical outcomes, shaping decision-making and public policies, and potentially exacerbating inequalities in healthcare.

Specifically, an important concern is the presence of sex and gender imbalances in collected human data, which might further compound and reinforce existing inequalities between sexes and genders in society. Taking as a reference the interpretation of the Office for National Statistics (ONS) and the UK government, biological sex refers to the “biological aspects of an individual as determined by their anatomy, a product of their chromosomes, hormones, and their interactions.” Whereas gender is a socio-cultural attribute that “is a personal, internal perception of oneself and so the gender category someone identifies with may not match the biological sex they were assigned at birth.”^4^ In June 2015, the National Institutes of Health (NIH) announced that sex as a biological variable must be part of research design and analysis in studies of vertebrate animals and humans (NOT-OD-15-102). Moreover, major granting agencies require sex and gender analysis to be integrated in research design^5^ although their inception followed different timelines (e.g., the European Commission in 2003, whereas the National Research Foundation of Korea in 2021). Despite such requirements, the quality of metadata linked to human data repositories is still inadequately addressed.^6^^,^^7^

In this article, we evaluate the current sex data imbalance in dbGaP and EGA by analyzing the existence or absence of such categories in the metadata of the submitted studies and samples. To assess sex imbalance and its changes over time, we begin by analyzing data gathered from 2018 onwards, marking the year the EGA policy was introduced. Subsequently, we incorporate all data collected since the inception of both databases. Additionally, we offer a series of recommendations based on our observations to encourage ethical methods in the gathering, sharing, and applying biological data.

## Results

### Sex balance in European Genome-phenome Archive and database of genotypes and phenotypes studies since 2018

Following the new metadata model introduced by the European Nucleotide Archive (ENA),^8^ the EGA repository mandated the sex category for all data submissions in 2018. On the other hand, dbGaP imposed such regulation in 2019 but only when submitting variants (VCF and PLINK files) data.^9^ Based on this difference between the two repositories, we compared the results of the two databases to assess the impact of these regulations. Thus, we collected the sex classification reported for the samples of the studies accumulated in EGA and dbGaP from 2018 up to the date of the data retrieval (November 2021). The sex classifications obtained align with three groups: female, male, and unknown. However, important details are lacking, such as (1) if experimental or other techniques were used to determine the sample’s sex, (2) if the reported sex is based on the assumed or recorded gender, and (3) the reasons behind the unknown category (e.g., inability to determine sex, confidentiality concerns, or donor’s unwillingness to disclose).

**Figure 1:**
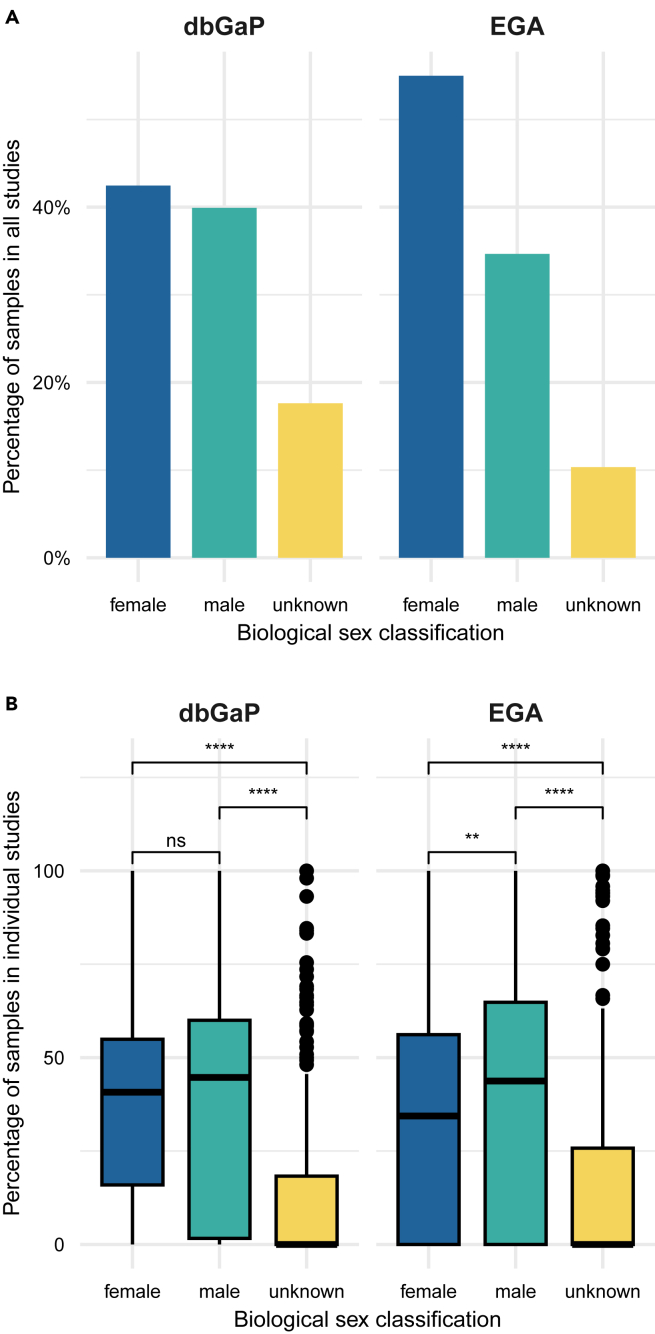
Distribution of samples’ classification of EGA and dbGaP since 2018 (A) Barplot depicting the distribution in the percentage of the sex classification in all the EGA and dbGaP samples (not unique) included in studies from 2018 to the date of data retrieval (November 2021). (B) Boxplot showing the samples’ sex classification distribution per individual study. The central line indicates the median, the box edges represent the interquartile range (IQR), and the whiskers extend to the minimum and maximum values within 1.5 times the IQR. A Wilcoxon statistical test was run to perform a paired comparison between the mean proportion of samples per study. ∗∗∗∗ means *p*-value <0.0001, ∗∗∗ pvalue <0.001, ∗∗ *p*-value <0.01, ns = not significant.

The majority of the samples in EGA (1,249,255 total samples over 1,490 studies) were classified as female (F = 55%), followed by male (M = 35%) and unknown (U = 10%) (Figure 1A). The number of female and male samples was more evenly distributed in dbGaP (42% female and 40% male), which comprised around 7 times as many samples (1,758,139) in half as many studies (750) as EGA. However, a higher number of the samples (18%) than for EGA fits the unknown category. It is important to note that the differences in the number of samples and studies between EGA and dbGaP cannot be attributed to any specific reason. We did not observe any preferential bias toward a particular type of study in either repository.

We conducted an analysis of the sex classification of samples within each study, comparing the counts of female, male, and unknown samples (Figure 1B). This approach offers an intra-study viewpoint, highlighting the distribution of male, female, and unknown samples within each study, rather than relying on aggregated totals. Our findings in dbGaP indicated a balanced representation of male and female samples at the study level, with a significantly low presence of samples with unknown sex. In contrast, data from EGA showed that, within individual studies, female samples were consistently underrepresented compared to male samples (Figure 1B). This detail is crucial because, although the overall cumulative count (Figure 1A) suggests a predominance of female samples, a closer examination at the study level revealed a tendency for female samples to be proportionally fewer than male samples. Similar to dbGaP, both female and male samples per study in EGA were significantly higher in proportion than unknown samples, indicating a comprehensive sex information recording.

**Figure 2:**
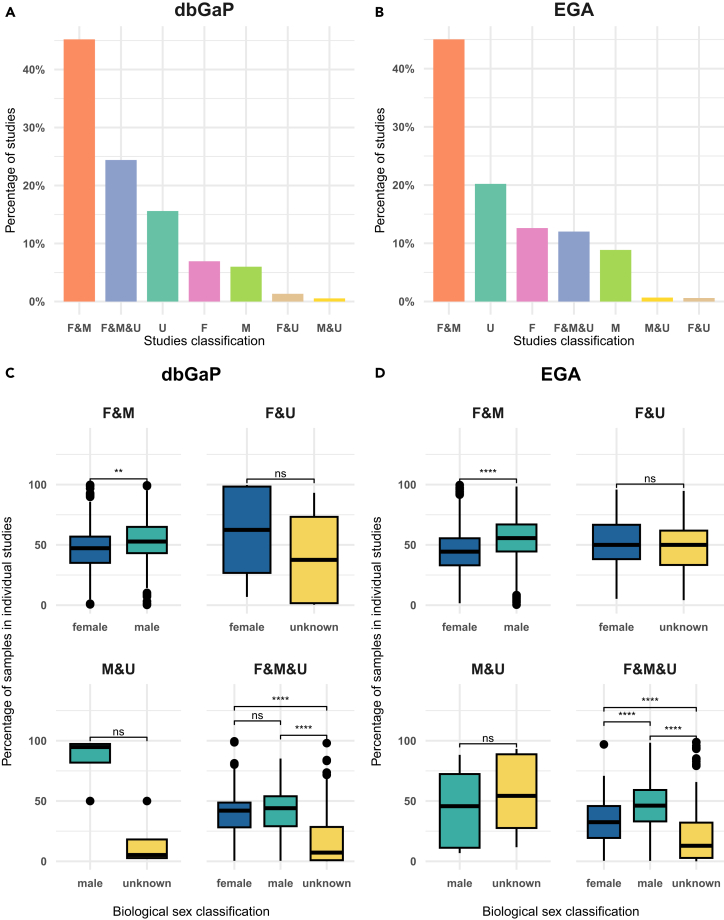
Distribution of studies classification of EGA and dbGaP since 2018 (A) and (B) dbGaP and EGA studies (2018–2021) classification respectively according to the sex representation of their samples. F, female; M, male and U, unknown. (C) and (D) samples’ sex classification distribution per individual study. The central line of the boxplots indicates the median, the box edges represent the interquartile range (IQR), and the whiskers extend to the minimum and maximum values within 1.5 times the IQR. A Wilcoxon statistical test was run to perform a paired comparison between the mean proportion of samples per study. ∗∗∗∗ means *p*-value <0.0001, ∗∗∗ p-value <0.001, ∗∗ *p*-value <0.01, ns = not significant. In the case of M&U for dbGaP, “ns” appears because no comparison was possible due to ties present in the data.

Next, we divided each study into categories based on the sex of their sample populations (Figures 2A and 2B). In both dbGaP and EGA, the majority of studies (45%, Figures 2A and 2B) used samples that were female and male (F&M). Studies with female, male, and unknown samples represent the second most common classification in dbGaP (F&M = 24%), while in EGA they represent the fourth type of most abundant studies (F&M = 12%). The second most common type of studies in EGA is those with unknown samples (U = 20%), a classification represented in 16% of dbGaP studies. We found that in both data repositories, there were more studies with only female (F) samples (7% in dbGaP and 12% in EGA) than with only male (M) samples (6% in dbGaP and 8% in EGA). Lastly, studies with female and unknown (F&U) or male and unknown (M&U) samples were always the least common in both repositories (F&U = 0.6% and M&U = 0.7% in EGA and F&U = 1.3% and M&U = 0.5% in dbGaP).

Similar to Figure 1B, we examined the distribution of samples per classification type at the study level (Figures 2C and 2D). In dbGaP and in EGA, there were significantly more male samples than females. Additionally, the combined F&M&U group showed a similar trend, with more female and male samples than unknown ones. Importantly, the representation of unknown samples in both F&U and M&U groups was high in both repositories, accounting for about half of the samples in the studies, with the exception of M&U comparison for dbGaP (4 studies only; statistical *p*-value could not be computed due to ties).

In conclusion, this analysis showed that it is not only important to quantify the general imbalances in the samples, but also their distribution within individual studies. EGA studies contained a lower number of unknown samples, mostly studied on their own, i.e., unknown-only studies. Male and female samples, however, were unequally distributed, with female samples being more prevalent at the general level and male samples at the study level. Nevertheless, the fact that both the biologically male and female sexes were represented in over half of the research is a positive finding. In contrast, although the representation of male and female samples in dbGaP was more balanced, there was a higher amount of unknown samples, which are usually included in studies that contain both male and female sample data. These results highlight the complexities and variations in sex representation within individual studies across the two databases. The discrepancies observed underscore the importance of considering study-level sex distribution, which may influence research outcomes and should be accounted for in data analysis and interpretation.

### Sex balance across time

**Figure 3:**
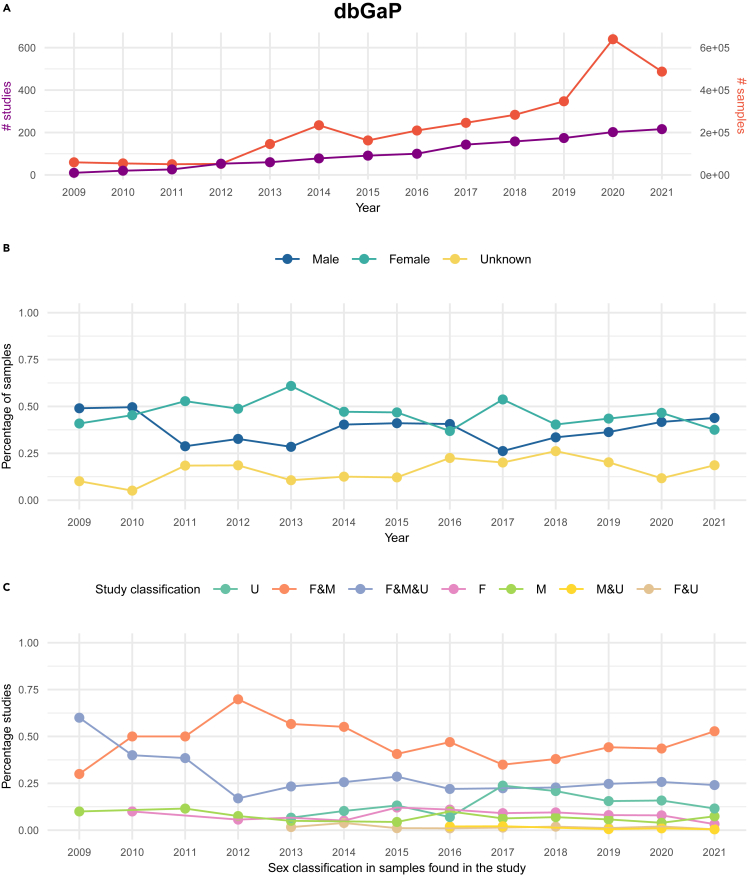
Quantification and sex classes’ distribution of dbGaP samples included in studies from 2009 to November 2021 (A) Represents the total number of dbGaP studies and samples used in dbGaP studies per year, (B) depicts the sex classification distribution in the percentage of the used samples in dbGaP studies yearly, and (C) shows studies classification according to the sex representation of their samples across time. F, female; M, male and U, unknown.

**Figure 4:**
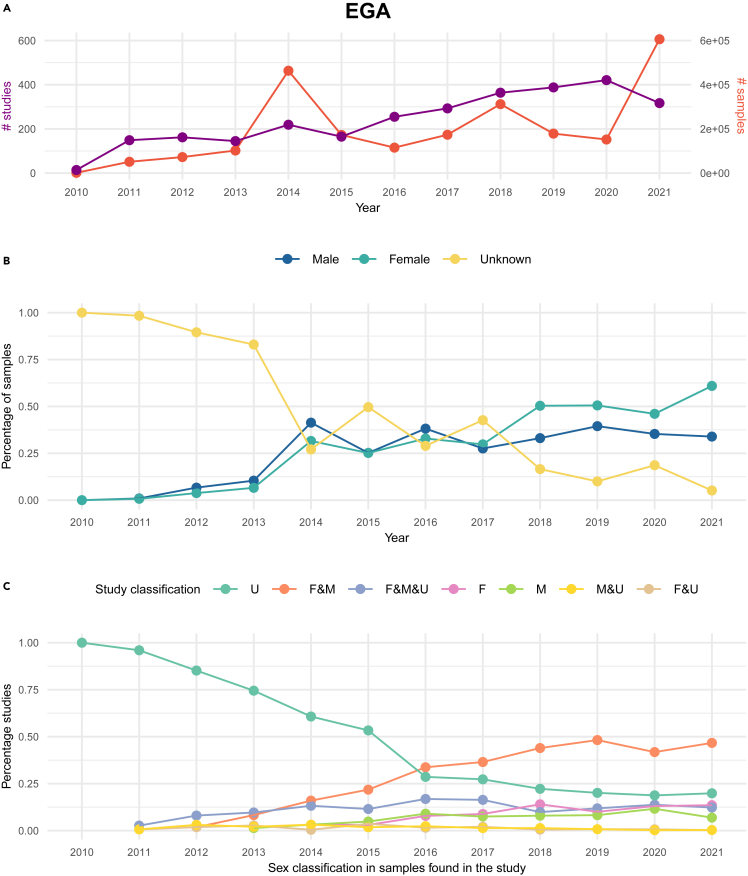
Quantification and sex classes’ distribution of EGA samples included in studies from 2010 to November 2021 (A) Represents the total number of EGA studies and the total number of samples used in EGA studies per year, (B) depicts the sex classification distribution in the percentage of the used samples in EGA studies yearly, and (C) shows studies classification according to the sex representation of their samples across time. F, female; M, male and U, unknown.

We expanded the prior study and analyzed EGA and dbGaP samples over time to have a better understanding of the evolution of female and male representation in research projects. In this way, we quantified the effect of the 2018 EGA policy and assessed how the sex classification of samples has changed over a longer time period. The collected data represented a period of over ten years, starting in 2009 for dbGaP and in 2010 for EGA, and they comprised a total of 1,337 dbGaP and 2,918 EGA studies, with 2,991,062 and 2,415,781 samples, respectively. Figures 3A, 4A, and S1 show the total number of studies over the years and the total number of samples included in each sex classification respectively. It is important to stress that diverse factors, which may not be fully traceable in a database’s historical records, may play an essential role in the variability observed in data collection trends, particularly in the early stages. These factors may comprise changes in metadata models, enhancements in portal usability, and the progressive adoption of the database and responsible best practices among the community.

We first analyzed the temporal sex classification distribution of dbGaP and EGA samples (Figures 3 and 4 respectively). Regarding dbGaP, the year 2020 has the highest number of submitted samples (639,969) (Figure 3A). Unlike other years, it coincides with one of the times with the lowest number of unknown classifications (Figure 3B). The previous also holds true when looking at the median number of unknown samples per F&M&U studies, meaning that a low number of unknown samples is a common feature among studies (Figure S2).

In EGA, the impact of the 2018 policy was evident as the proportion of unknown samples significantly decreased compared to female and male samples for the first time (Figure 4B). From 2010 to 2013, over 75% of samples were categorized as unknown, a fact that cannot be justified by sex-related phenotypes (Figure S3). This percentage dropped to 50-25% in 2014 and remained comparable to male and female sample proportions for four years. Although we lack access to historical submission guidelines, we speculate that this reduction in the unknown category is likely attributed to changes in sample metadata submission requirements introduced in July 2013 (internal communication). These findings underscore the importance of policy updates in enhancing data reporting and transparency in the EGA repository.

Since 2018, and similar to dbGaP, EGA unknown samples represented less than 25% of the samples, with the difference that it reached a minimum of 5% in 2021. Since 2010, there was an equal distribution of male and female samples; however, this was no longer the case as of 2017, since we consistently observed more female samples being used in EGA studies than male ones (Figure 4B). In the case of dbGaP, the percentage of female and male samples is comparable in most of the years, and more female than male samples are observed in the years 2011–2013 and in 2017. Unlike in EGA, unknown samples in dbGaP positively remain lower than 25% throughout all the years. However, there seems not to be any decreasing pattern at any point, suggesting that implementing a dedicated policy to control these types of samples could be beneficial in the future.

Next, we analyzed EGA and dbGaP studies according to the sex classification of their samples across time (Figures 3C and 4C respectively). Regardless of the year, while between 20% to almost 75% of the dbGaP studies were always represented by F&M and F&M&U classifications, the rest of the study classifications (F, M, U, F&U, and M&U) were never superior to 25% of the total (Figure 3C). We want to highlight that the F&M&U category masks a problem of unlabeled data as the median proportion of samples per study was constant throughout the years (Figure S2). While in EGA, the median number of unknown samples per study dropped over time, especially after 2015, it is not the case for dbGaP, which while low, there is no observed pattern.

Regarding EGA, 100% of the studies contained only unknown samples (U) in 2010 (Figure 4C). This value gradually decreased, reaching its minimum (∼20%) in 2021. Overall, a statistically significant decreasing trend in EGA unknowns is noted (Mann-Kendall trend test: *p*-value = 8.30e-06, Z-value = −4.457). In contrast, no trend is observed in the corresponding dbGaP series (Figure 3B) (Mann-Kendall trend test: *p*-value = 0.058, Z-value = 1.891). More information on this trend analysis is reported in Methods S1 and Figure S4. In EGA, after 2013, at the same time that the unknown-only studies decreased, mixed studies increased. F&M studies began to outnumber all other types in 2016, reaching their peak in 2019 and 2021 when they accounted for about 50% of all studies. It seems that regardless of the 2018 policy, there was already a good practice of not performing studies with unknown-only samples, especially since 2016, which is likely further benefited by the new policy.

In summary, we were able to identify sex representation imbalance in the EGA and dbGaP repositories by quantifying data changes across time. From our results, we conclude that the EGA 2018 policy has evidenced that a change indeed is possible in the sex reporting of samples as we observed a drastic reduction in the number of unknown samples. In contrast, by grouping the studies according to their sex representatives (for example, F&M&U) rather than analyzing all of them together, we revealed an alarming constant presence of samples classified as unknown in recent years in both repositories.

### Misrepresentation of one of the sexes in studies is not always justified

In addition to the absence of sex information in biological samples, studies that only take into account one of the two biological sexes could represent a potential source of bias if the resulting conclusions are applied to the entire population. In light of this, we evaluated which phenotypes are more prevalent in females compared to male populations in the examined studies.

**Figure 5:**
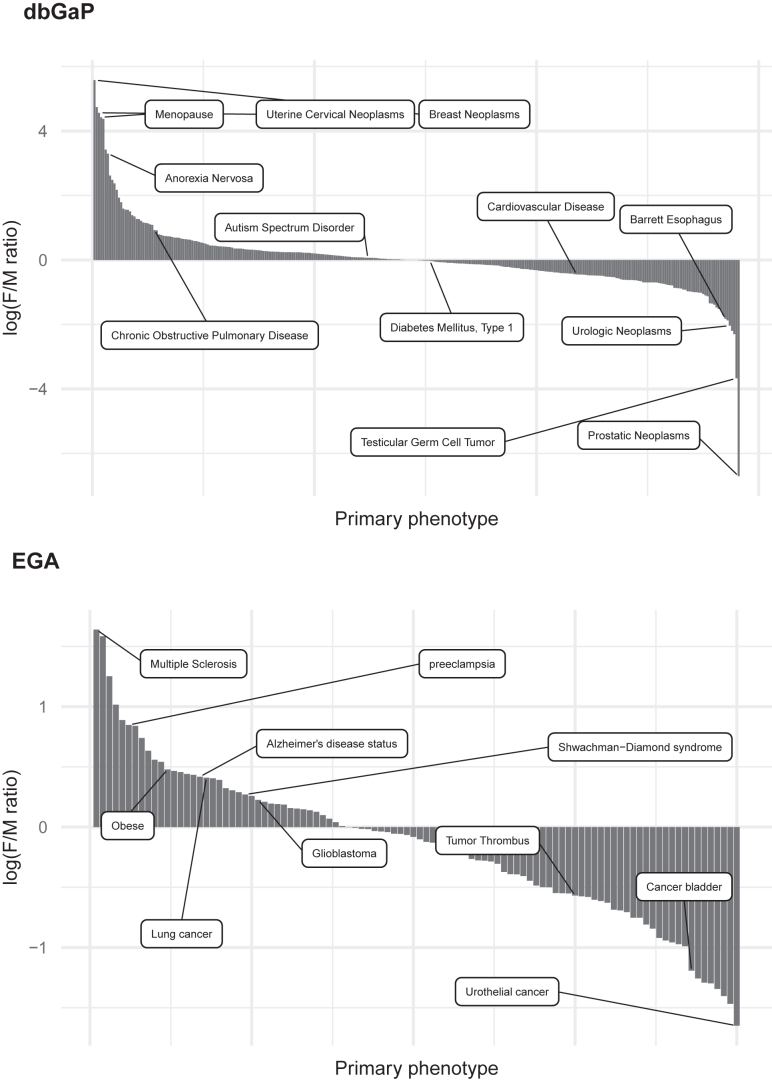
Female to male ratio of phenotypes in dbGaP and EGA studies from 2018 to 2021 All phenotypes in the selected studies are included in the figure for dbGaP while only the top 100 phenotypes after ranking studies by the number of samples are considered in the case of EGA.

Figure 5 presents the female-to-male ratio per phenotype, focusing on EGA and dbGaP data from 2018 onwards. To calculate this ratio, we analyzed each study identifier individually and determined the mean ratio when multiple studies corresponded to the same phenotype. The full table is in Table S1. One limitation is that while dbGaP phenotypes are more homogeneously represented in the form of “primary phenotype,” in the case of EGA this information is provided as a free text variable. Therefore, in the case of dbGaP, available phenotypes from all studies are considered (*n* = 395; 38 studies out of 750 do not report the phenotype) while in the case of EGA, only the top 100 phenotypes with more samples have been manually curated.

In both repositories, we observed sex-specific phenotypes, such as menopause (83.6 F to M ratio) or prostatic neoplasm in dbGaP (0.001 F to M ratio) or preeclampsia in EGA (2.33 F to M ratio), present in both males and females, which made us hypothesize that either subject may have been classified by gender instead of biological sex or the study design includes samples of the other sex for given reasons. As stated above, we have no way of knowing whether the classification was based on sex or gender and it reinforces the need for a clear definition of sex and gender in biological databases.

A higher number of female samples with anorexia nervosa and chronic obstructive pulmonary disease (COPD) were studied in dbGaP, while a higher number of female samples with multiple sclerosis and obesity were studied in the EGA repository. In the case of male samples, diseases such as cardiovascular disease and Barrett’s esophagus were more studied in dbGaP and bladder cancer, and urothelial cancer in the case of EGA. Overall, if there is more incidence of a disease among a certain population, such as females or males, it is expected that more samples of that population will be studied.

However, some diseases can affect both female and male populations and only one sex is represented in the study. Therefore, we evaluated if there is a valid biological reason for the absence of samples from one sex in the studies of a specific phenotype in either of the two databases.

We selected studies that contained only *female or male s*amples and at least 100 samples. 45 out of the 110 selected studies (dbGaP = 22 and EGA = 23) contained only male samples and the rest (dbGaP = 17 and EGA = 48) only female samples (Table S2). Among those, we manually identified 25 studies (6 from dbGaP and 19 from EGA) where the phenotype is examined only in one sex (Table 1), although the phenotype can affect both sexes. We observed discrepancies in the classification of samples reported for two EGA studies (EGAS00001002941 and EGAS00001005323) compared to what is presented in the respective publications. These discrepancies are concerning, and they underscore the importance of conducting thorough quality checks when utilizing metadata of this nature.

**Table 1:** Selection of EGA and dbGaP only-female or only-male studies of phenotypes with incidences in the entire human population

Repository	Study identifier	Phenotype	Sex label	Total Samples	Title
dbGaP	phs001258.v2.p1	MicroRNAs	M	676	Total exRNA Profiles from Plasma, Saliva, and Urine of Healthy Subjects
dbGaP	phs001500.v2.p1	Melanocytes	M	106	National Cancer Institute (NCI) Primary Human Melanocyte QTL Study
dbGaP	phs002226.v1.p1	HIV	M	3747	Multicenter AIDS Cohort Study (MACS)
dbGaP	phs002295.v1.p1	Carcinoma, Non-Small-Cell Lung	M	234	Cross-site Concordance Evaluation of Tumor DNA and RNA Sequencing Platforms of CIMAC-CIDC Network
dbGaP	phs001916.v2.p1	Healthy Aging	F	100	Pilot Sequencing Study: DNA Hydroxymethylation and Gene Expression in Peripheral Blood Mononuclear Cells in Healthy Human Aging
dbGaP	phs002366.v1.p1	Lung Neoplasms	F	1195	Lung Cancer Genetic Study among Asian Never Smokers
EGA	EGAS00001003221	Circulating microRNA	F	198	Temporal stability of circulating microRNAs in human serum
EGA	EGAS00001003242	Colorectal cancer	F	107	Genomic DNA of tumor tissues, adjacent normal tissues, and peripheral blood were extracted using QIAamp DNA mini Kit (QIAGEN, cat. #51306)
EGA	EGAS00001003258	Circulating DNA in cancers	F	118	Enhanced detection of circulating tumor DNA by fragment size analysis
EGA	EGAS00001003705	Non-small-cell lung adenocarcinoma	F	224	Multi-layered molecular characterization defines prognostic subtypes of lung adenocarcinoma in Asian never-smokers
EGA	EGAS00001003789	Non-small-cell lung adenocarcinoma	F	220	“KOREAN” never-smoker female adenocarcinoma RNA-seq
EGA	EGAS00001004900	Human regulatory T cells	F	158	Single-cell chromatin accessibility landscape identifies tissue repair programs in human regulatory T cells
EGA	EGAS00001004994	Immune-mediated aplastic anemia	F	192	Somatic mutations in lymphocytes in patients with immune-mediated aplastic anemia
EGA	EGAS00001005517	Celiac disease	F	1440	Differential expression profile of gluten-specific T cells identified by single-cell RNA-seq
EGA	EGAS00001005323	Fetal aneuploidy detection	F	1092	Systematic evaluation of NIPT aneuploidy detection software tools with clinically validated NIPT samples
EGA	EGAS00001002941	Lung Adenocarcinoma	M	678	The genomic landscape of lung adenocarcinoma in East Asians
EGA	EGAS00001002965	Common SNP variations	M	140	SNP array data for 140 individuals from 5 populations in Pakistan
EGA	EGAS00001003293	Cell cycle in human iPSCs	M	192	Pseudotime ordering of cell cycle state
EGA	EGAS00001003501	Immune cell response	M	183	Chromatin accessibility in cytokine induced immune cell states
EGA	EGAS00001003653	DNA methylation	M	120	Indonesian methylation data
EGA	EGAS00001003671	Transcription	M	179	Indonesian RNA-seq data
EGA	EGAS00001003823	Immune cell response	M	141	Gene expression regulation in cytokine induced immune cell states
EGA	EGAS00001004192	Transcription	M	977	Transcriptomic response of miRNAs of monocytes to bacterial and viral stimuli assessed by RNA-seq in Africans and Europeans
EGA	EGAS00001004758	Idiopathic Pulmonary Fibrosis	M	291	The transition from normal lung anatomy to minimal and established fibrosis in Idiopathic Pulmonary Fibrosis
EGA	EGAS00001005204	Hepatocellular carcinoma	M	146	Dissecting the Spatial Heterogeneity of Single Circulating Tumor Cells in Hepatocellular Carcinoma

Phenotypes are sourced from the primary phenotypes field in dbGaP and curated manually in EGA (refer to the STAR Methods section, "data and code availability"). EGAS00001005323 and EGAS00001002941 are studies in which EGA metadata classification differs from that reported in the results of the associated publications.

For instance, the EGA study “The transition from normal lung anatomy to minimal and established fibrosis in Idiopathic Pulmonary Fibrosis (IPF)”^10^ considers only male samples, although IPF has comparable disease progression and survival rates for both.^11^ Likewise, the dbGaP study “National Cancer Institute (NCI) Primary Human Melanocyte QTL Study,”^12^ only contains males given that this disease is more prevalent among this population^13^ although it still affects part of the female population^14^ (https://seer.cancer.gov/faststats). It is important to emphasize that, in precision medicine, studying sex-specific cohorts is vital. While authors of publications related to the previous study (phs001500.v2.p1) and study phs001916.v2.p1 (preset in Table 1) did report their study limitations due to the unilateral consideration of either female or male subjects, the scientific community should advance by investing in conducting comparable sex-specific studies to fill the current gaps in these databases, for instance, exploring IPF in female smokers and melanoma in females.

To complement this work, we conducted a survey (Document S2) concerning sex reporting in human databases and related aspects, among the participants of the ELIXIR BioHackathon Europe 2021 (Figure S5). The BioHackathon is an annual event that brings together bioinformatics experts, software developers, and researchers, organized by ELIXIR, an international organization that aims to facilitate access to and the use of bioinformatics resources across Europe. Despite the survey’s small participant size (*n* = 66, Table S3), and with 64% of the respondents from Spain, it exposed a significant lack of basic knowledge on the topic among those who are more exposed to interacting with these databases at professional level. Although nearly 70% of the respondents considered it important to include diversity in scientific publications as well as in financing processes (question 10, Figure S9), 67% of all participants were unaware of the ethical guidelines or international reference manuals regarding the inclusion of sex and gender diversity in science (question 4, Figure S10). More information on such limited yet, in our view, insightful qualitative analysis can be found in Methods S2.

In summary, we identified instances where diseases affecting both sexes were studied in only one sex, prompting questions about the validity of such disparities in relation to the biological basis of these diseases. We also revealed discrepancies in the classification of samples for specific phenotypes, particularly in two EGA studies, when compared to their respective publications. These discrepancies raise concerns about the accuracy and consistency of metadata classification. Our findings underscore the importance of rigorous quality checks when utilizing metadata and the need for standardized approaches to address sex imbalance in biomedical research, ensuring more accurate and representative outcomes.

## Discussion

We analyzed the impact of the 2018 EGA policy in comparison to a less regulated database such as dbGaP in order to assess to what extent this measure can impact positively data sex classification. As shown in Figure 1, EGA reduced unknown samples after 2018, but female samples dominated generally, while male samples were more common within individual studies. In dbGaP, male and female sample representation was balanced, but unknown samples were present in studies with both sexes. Regarding studies classification by sex, notably, both biologically male and female sexes were represented in over half of the research, reflecting an encouraging pattern (Figure 2B). Similar results are observed for dbGaP, with balanced male and female sample representation (Figures 1A, 1B, and 2A).

From a temporal perspective, our main finding suggests that EGA’s policy is successful in reducing the "unknown" sex classification in datasets (Figures 4B and 4C). This accomplishment is likely facilitated by the implementation of progressive practices aimed at diminishing unknown samples, especially after 2013. This practice is clearly not perceived in dbGaP studies (Figures 3B and 3C). Complementarily, our study aimed to assess the impact of EGA’s qualitative policy implementation on encouraging the systematic reporting of sex and gender categories in biomedical data repositories. Conducting an anonymous survey (Table S3) to assess experts' knowledge and attitudes regarding these aspects, we emphasized the need for ongoing training, resources, and guidelines to improve data completeness and ethical inclusion.

Based on our results, we propose a set of recommendations to improve biomedical research and practice, fostering representativeness in the data, social inclusion, and governance. These actions are expected to have an impact on the biomedical research community as well as other stakeholders such as governments, policymakers, and the society:

1. **Provide clear definitions of sex and gender.** According to the Gendered Innovations report led by L. Schiebinger,^15^ *sex* refers to the biological characteristics of both humans and animals while *gender* is associated with sociocultural attitudes, behaviors, and identities. Analyzing the webpages of EGA and dbGaP, we observed a lack of consistency in the definition of these concepts and they are often mixed (for instance, EGA stores only the “gender” field but is associated with the biological characteristic, i.e., “female,” “male,” or “unknown”). The perpetuation of such practices leads to confusion and directly affects the quality of the data, particularly the accuracy, reliability, and validation. In some cases, sex and gender are used interchangeably due to a lack of details on how the information is defined in the original sources. For example, in Figure 5, we analyze F/M ratios in studies without the ascertainment of whether the reported "sex" was defined by chromosomes or assumed from gender or sex assigned at birth. Additionally, we must also acknowledge the existence of intersex individuals, whose biological characteristics may not fit within the traditional binary categorizations of male or female or changes in legal documentation for transgender individuals, consequently impacting data reporting. To mitigate this, these repositories should consider extending the options of categories and avoiding free text to enhance dataset accuracy and consistency.
2. **Improve data management.** Over the last few years, it has become mandatory to fill the sex field when creating datasets or adding new inputs. However, as observed in the results of Figures 1, 2, 3, and 4, this is not enough as this field is often filled as "unknown." To ensure data quality and interoperability, standardization efforts should begin during the design phase, including the use of limited checkbox options, adherence to legal data regulations, and the adoption of common data models.^16^ Centralizing data in a single repository further streamlines processes, addressing issues related to data submission and analysis, as discussed in the study’s limitations.
3. **Privacy preservation.** In the biomedical field, part of the data that is being used is considered sensitive according to the GDPR. Today privacy is preserved through various techniques such as data anonymization, encryption, and secure data-sharing protocols.^17^ As privacy-preserving technologies evolve, they could enable more secure and confidential collection and analysis of sensitive data related to sex.^18^ This could influence the development of more effective policies and interventions to address sex reporting imbalances while ensuring data protection and privacy compliance. For instance, when individuals are assured that their privacy is upheld, they are likely to be more forthcoming in accurately disclosing their sex, rather than opting for "Refused to tell," resulting in more reliable data. Similarly, conducting re-analyses of karyotypes with explicit individual’s consent, while ensuring privacy protection, can address issues of underreporting. Anonymization and other techniques are put in place to enhance user trust and public engagement. However, it is worth considering whether these anonymization processes contribute to the occurrence of unknown sex classifications. Therefore, studies with unreported metadata should be categorized as either patient anonymous or not, enabling a more transparent approach to handling cases where the subject sex is undisclosed. Interestingly, some works are putting efforts into studying the relation between biological sex or ethnicity with genomic data. For example, ethnicity and biological sex may be predictable from the genomic data gathered (e.g., dbGaP uses an automated tool called GRAF to check if the annotations in the data match the predicted annotations^19^).
4. **Transparency and accountability** are essential to foster open science, to reduce the cost of creating and curating datasets, and to enhance the quality of the datasets and the whole pipeline. This concern is highlighted by the discrepancies in sample classification outlined in Table 1, particularly for two EGA studies (EGAS00001002941 and EGAS00001005323), when compared to information presented in their respective publications. These discrepancies underscore the significance of rigorous quality checks when working with such metadata. Ensuring accurate reporting of sample sex metadata entails not only establishing clear definitions of sex and gender but also specifying the method used for reporting it. For instance, specifying the type of sex reported—whether based on chromosomes, genes, hormones, reproductive anatomy, secondary sex characteristics, and so forth—would significantly enhance accuracy and consistency in data interpretation. In this regard, metadata models and ontologies play a key role in standardizing sex and gender reporting. However, we observe discrepancies that currently hinder the realization of such best practices. For instance, the OMOP Common Data Model (CMD) blends biological sex considerations under the Gender domain (https://www.ohdsi.org/web/wiki/doku.php?id=documentation:vocabulary:gender), while the Gender, Sex, and Sexual Orientation Ontology (GSSO)^20^ clearly distinguishes between sex (http://purl.obolibrary.org/obo/NCIT_C28421) and gender (http://purl.obolibrary.org/obo/NCIT_C17357) definitions and subcategories. Hence, transparency and proper standards should be guaranteed across the dataset’s life cycle and used as a means to prevent any misuse. Again, standard processes are not yet defined although there are initiatives such as Healthsheets, which aim to help dataset creators and consumers improve self-reflection, documentation, and decision-making.^21^
5. **Promote education and social impact assessment.** It is fundamental to raise awareness on the importance of depositing good quality metadata in biomedical repositories to study how technology will benefit society, which requires higher standards of education and social impact awareness for both data providers and database administrators, and designers. According to Schiebinger,^15^ it is necessary to fix the knowledge to stimulate more responsible science and technology, being biomedical metadata reporting is an example not to be overlooked. This should be adopted from an early stage, in educational strategy and material that includes a sex and gender perspective. Include participatory methods to involve underrepresented communities or end users affected by the technology in the process. Following European recommendations on responsible research, a multidisciplinary team could also foster diversity, inclusiveness, and hence, fairness. This is especially relevant in careers such as medicine, biomedicine or computer science, and artificial intelligence, but it should also be enhanced by training in companies or research institutions, and so forth. In this regard, it is the duty of researchers, universities, funding agencies, and editors to put an end to unwarranted scientific practice.^22^

Sex and gender dimensions have historically been overlooked in scientific research investigations, leading to unintentional and sometimes unnoticed imbalances in research outcomes.^22^ More recently, the successful application of AI to the health domain, and particularly, in precision medicine, is a major achievement in science and technology.^23^ AI for health is particularly thriving in the domains of medical imaging and digital medicine, with successful applications in digital pathology^24^ and wearable technologies.^25^ The quality and content of these data have an immense impact on what and how AI learns. The use of AI can produce discriminatory results and spread them throughout society if the data have biases, such as an inaccurate representation of some demographic groups or missing data. Concerns about the risk of AI incurring ethical issues have been recently pointed out, particularly in relation to sex and gender bias in AI.^26^ Collectively, our findings and recommendations emphasize the multifaceted nature of sex representation within individual studies across the two databases and highlight the significance of considering study-level sex distribution, which can profoundly impact research outcomes and should be duly considered in data analysis and interpretation.

This study, which stemmed from our voluntary participation at the ELIXIR BioHackathon 2021, exemplifies our commitment to addressing these data imbalances in biological databases. We believe our work contributes to the standardization of such efforts, fostering research integrity. As we look ahead, the focus remains on rectifying imbalance, promoting inclusion, and ensuring a more equitable future in research.

### Limitations of the study

Our study has certain limitations that warrant consideration. First, we relied on EGA data providers, participating as authors of this article, for downloading metadata. Second, the EGA phenotype reports are presented in a free-text format, which complicates efforts to standardize and compare them with dbGaP phenotypes, potentially introducing variability in our analysis.

Additionally, our analysis revealed instances where the reported classification of sex in the data did not perfectly align with the information later found in the associated publications. However, it is important to note that we could not systematically verify the extent of these discrepancies, as manually reviewing all publications from all included studies was not feasible and several publications are not open access. Therefore, our reference for sex classification primarily relies on the available data, with the assumption that any such discrepancies are relatively minor. These limitations underscore the importance of cautious interpretation when using the data and metadata from EGA and dbGaP in our study.

Lastly, one of the study limitations pertains to the time frame and nature of the BioHackathon event in which the qualitative analysis was conducted (Methods S2). The BioHackathon Europe, organized by ELIXIR yearly, gathers bioinformaticians from across the globe for an intense week of hacking on diverse and exciting projects. This event comprises five days of collaborative coding to address bioinformatics challenges. The condensed and voluntary nature of this event imposed constraints on the depth and scope of the qualitative analysis performed *in situ*.

## Resource availability

### Lead contact

Requests for further information and resources should be directed to the lead contact, Davide Cirillo (davide.cirillo@bsc.es).

### Materials availability

This study did not generate reagents, cell lines, or any biological material.

### Data and code availability

• EGA and dbGaP extracted quantitative data have been deposited at Zenodo and are publicly available as of the date of publication. Accession number is listed in the key resources table.
• All original code has been deposited at Zenodo and GitHub and is publicly available as of the date of publication. DOIs are listed in the key resources table.
• Any additional information required to reanalyze the data reported in this article is available from the lead contact upon request.

## Acknowledgments

The work has been supported by Bioinfo4Women through the project Excelencia Severo Ochoa (ref. CEX2021-001148-S) and the European Commission's Horizon 2020 Program, H2020-SC1-DTH-2018-2020, “iPC - individualizedPaediatricCure” (GA 826121). This work was conceptualized and prototyped during the BioHackathon Europe, organized and funded by the ELIXIR Hub in November 2021 in Barcelona. We thank the organizers for an opportunity to participate in such a productive and collaborative event. The authors would like to acknowledge the initiative Bioinfo4Women, Laura Rodríguez Navas (Spanish National Bioinformatics Institute, INB/ELIXIR-ES and 10.13039/501100006433Barcelona Supercomputing Center, BSC), Eva Alloza (Spanish National Bioinformatics Institute, INB/ELIXIR-ES and 10.13039/501100006433Barcelona Supercomputing Center, BSC), Francisco Garcia-Garcia (Prince Felipe Research Center, 10.13039/501100024789CIPF), Babita Singh (Center for Genomic Regulation, 10.13039/100017169CRG), Ben Busby (DNANexus), and Michael Feolo (dbGaP) and the NCBI dbGaP support team. C.P. is supported by the fellowship Juan de La Cierva - Formación from the Spanish Ministry of Education and Science (ref. FJC2021-046655-I).

## Author contributions

D.C. and N.B. conceived of the project and participated in its design and the coordination of the quantitative and qualitative analysis, respectively. A.C. and G.L.H. contributed to the qualitative analysis. V.R.S., O.P., M.M., D.S., C.P., and A.M.A., contributed to the quantitative analysis. V.R.S. took care of optimizing the content of the article, including figures, text, supplemental material and scripts. A.J., M.M., J.R. provided support regarding EGA. A.V. and M.J.R. supported the project. All authors participated in the ELIXIR Biohackathon Europe 2021 and contributed to the final version of the article.

## Declaration of interests

A.J., M.M., J.R. develop, maintain and coordinate the EGA database. D.S., A.M.A., and G.L.H. work for private companies.

## STAR★Methods

### Key resources table

**Table:** 

REAGENT or RESOURCE	SOURCE	IDENTIFIER
**Deposited data**		

EGA and dbGaP data	This paper	https://zenodo.org/records/13350832

**Software and algorithms**		

Metadata Download and Figures’ Code	This paper	https://zenodo.org/records/13350896
GitHub Repository	This paper	https://github.com/social-link-analytics-group-bsc/biohackathon-project-35

**Other**		

Metadata Distribution Guidelines	EGA	https://ega-archive.org/access/download/metadata/
Subject Sample Telemetry Report (SSTR) API	dbGaP	https://www.ncbi.nlm.nih.gov/gap/sstr/swagger/
BioHackathon Europe 2021 website	Elixir Hub	https://2021.biohackathon-europe.org/

### Method details

#### The ELIXIR Biohackathon Europe 2021

This work is the result of the project “*FAIRX: Quantitative bias assessment in ELIXIR biomedical data resources*'' that was developed by the authors at the ELIXIR BioHackathon Europe (BH2021) hosted in Barcelona in November 2021 (https://2021.biohackathon-europe.org/). We performed a quantitative analysis to measure the data quality and find possible imbalance in sex reporting that might lead to bias in two large and well-known human data repositories, EGA and dbGaP. Additionally, we performed a qualitative analysis (Methods S2) to assess the awareness about such issues among the participants of the BH2021 and the Bioinfo4Women Twitter account (https://twitter.com/bioinfo4women) by submitting a questionnaire during the days of the event and a qualitative review of all the procedures and requirements by dbGaP and EGA metadata repositories.

#### EGA data retrieval and processing

We extracted any mention of the ‘sex’ category in the metadata associated with the samples used in all the studies accumulated in EGA. This data can be accessed through the EGA public metadata API. The sex variable had four possible values: male, female, unknown, and null. We counted unknown and null together under the category ‘unknown’. The filters for EGA data were study objects from 2018 to the date of download (November 2021), public on the webpage (status = RELEASED), linked to sequencing data. Since 2018, the European Genome-phenome Archive (EGA) registered a total of 1502 studies as of November the 10th 2021. From these studies, 1489 are linked to sequencing data and the rest are linked to array data, which are not queryable through the public metadata API. For this reason, we only considered the 1489 EGA sequencing studies in this analysis. The selected studies contain a total of 705502 unique samples, 4431 of them are from before 2018. In those cases, when a sample has no sex classification, it is considered as *unknown*.

For Figure S3, phenotypes from 2010 to 2013 EGA studies with unknown-only samples were extracted using the txt2hpo python package (https://github.com/GeneDx/txt2hpo) which, based on text, predicts the Human Phenotype Ontology^27^ (HPO) ID. We used the “description” text field from EGA’s studies metadata as input for txt2hpo to retrieve the related HPO IDs and we used the most recent hp.obo file (v2023-07-21) to extract each HPO IDs’ terms. The rest of phenotypes used in Section “Misrepresentation of one of the sexes in studies is not always justified” were provided by the EGA team. The file containing the resulting data can be found in the data and code availability section.

#### dbGaP data retrieval and processing

During the ELIXIR BioHackathon Europe 2021, we accessed dbGaP metadata through their public FTP access (https://ftp.ncbi.nlm.nih.gov/dbgap/studies/). We collected the counts of the reported sex category in the samples used in their studies by parsing through their XML files. We observed that the formatting of the metadata was not consistent across all the XML files (e.g. “gender” is sometimes used to refer to sex) and, thus, their processing required careful handling. In May 2024, we used the newly developed API system, where metadata is better structured and easily accessible. We used the dbGaP Subject Sample Telemetry Report (SSTR) API (https://www.ncbi.nlm.nih.gov/gap/sstr/swagger/) and re-downloaded the metadata for the same initial set of dbGaP studies downloaded in 2021. All scripts used for data retrieval and processing are available on GitHub for transparency and reproducibility.

#### Questionnaire about sex reporting in databases

An anonymous survey (Table S3) with 18 questions (7 of which were designed to obtain socio-demographic data) was distributed among Biohackathon ELIXIR 2021 participants as well as through social networks such as the official Twitter account of the Bioinfo4Women initiative from the Barcelona Supercomputing Center (https://cutt.ly/qN2iG0Z). Additionally, at the qualitative level, we reviewed all the available documentation from dbGaP and EGA platforms and analyzed which variables, terms and concepts were obligatory in the submission process or in the guidelines and when such changes were introduced.

### Quantification and statistical analysis

Statistical analyses were performed to assess the sex classification distributions within the EGA and dbGaP repositories. Data were quantified in terms of the proportion of samples classified by sex (female, male, unknown). The primary statistical test used was the Wilcoxon paired test to compare mean proportions of samples per study. This statistical test was chosen because it is a non-parametric test that does not assume a normal distribution of the data, making it suitable for the small sample sizes and potential non-normality in the sex classification data. Significance levels were indicated as follows: ∗∗∗∗ for p < 0.0001, ∗∗∗ for p < 0.001, ∗∗ for p < 0.01, and ns for not significant. Specific statistical details, including the number of samples and studies, are presented in the figure legends and results sections. Additionally, the Mann-Kendall trend test was applied to evaluate temporal trends in the data, specifically to detect monotonic trends in the classification of sex over time. All details of the analysis are provided in Methods S1.

## Supplemental information

Document S1. Figures S1–S15 and Methods S1 and S2

Table S1. Female to male ratio of phenotypes, related to Figure 5 Female to male ratio per phenotype in EGA (only top 100 phenotypes) and dbGaP data from 2018 onwards.

Table S2. Gender-exclusive studies with over 100 Samples, related to Table 1 Full list of studies that contained only female or male samples and at least 100 samples in EGA and dbGaP studies from 2018 to 2021. Highlighted in bold are studies for which single gender analysis is not justified based on the phenotype.

Table S3. Qualitative questionnaire responses and harmonized data, related to Figures S5–S15 Excel file containing the responses of the qualitative questionnaire. The table contains two related tabs: “ST3A” tab contains the raw questions and responses; “ST3B” tab contains the corresponding processed responses for harmonization and representation purposes”.
