# Supplementary material for: Analyzing sex imbalance in EGA and dbGaP biological databases: Recommendations for better practices
Source: iScience. 2024 Sep 23;27(10):110831. doi: 10.1016/j.isci.2024.110831 (PMC11519560; doi:10.1016/j.isci.2024.110831)
Supplement: Document S1. Figures S1–S15 and Methods S1 and S2 [file mmc1.pdf]

iScience, Volume *27*

Supplemental information

Analyzing sex imbalance in EGA and dbGaP biological databases: Recommendations for better practices

Victoria Ruiz-Serra, Nataly Buslón, Olivier R. Philippe, Diego Saby, María Morales, Camila Pontes, Alejandro Muñoz Andirkó, Gemma L. Holliday, Aina Jené, Mauricio Moldes, Jordi Rambla, Alfonso Valencia, María José Rementeria, Atia Cortés, and Davide Cirillo

Supplemental Figures

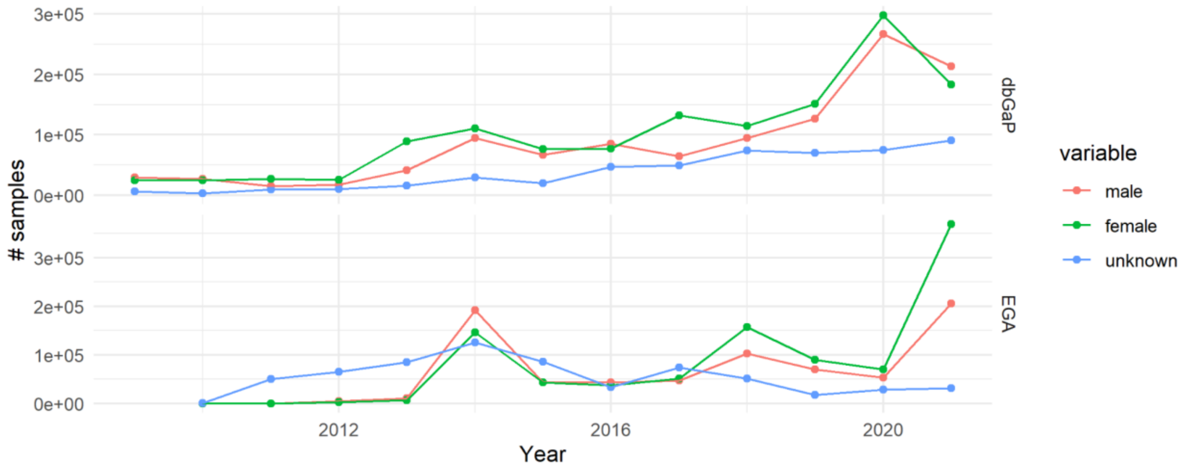

Figure S1. Overview of samples sex classification distribution in EGA and dbGaP repositories across time. Line plot representing the total number of samples per year and by sex classification (male, female or unknown), submitted to dbGaP (upper panel) and EGA (lower panel) repositories between 2010 and 2021, both included.

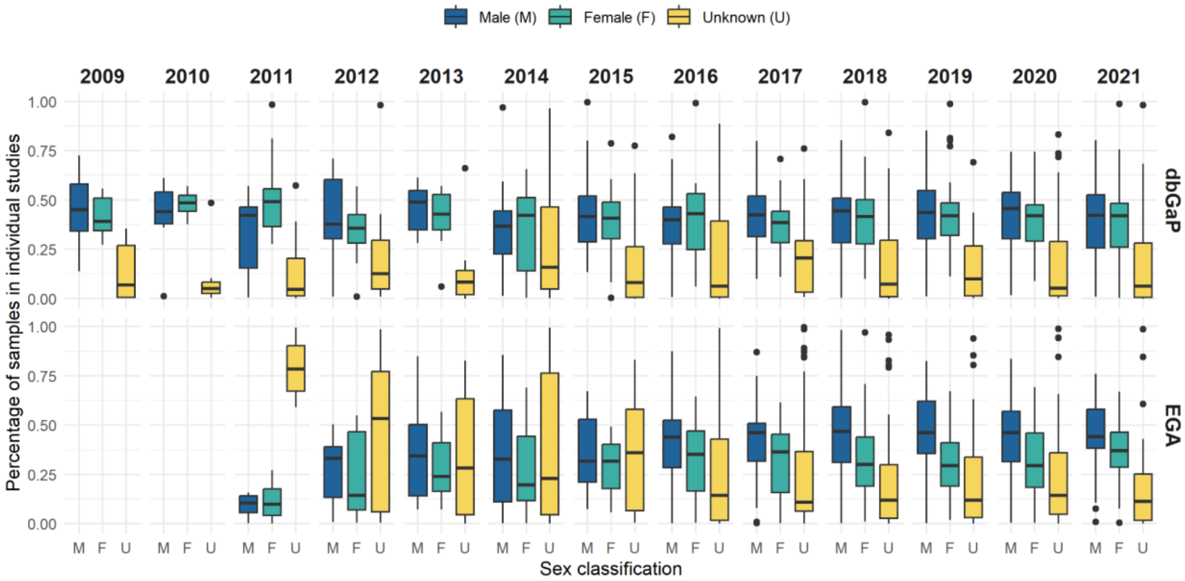

Figure S2. Trend of samples' sex classification across time in *F&M&U* studies. Boxplot showing the sex classification distribution of samples per study in EGA and dbGaP ***F&M&U*** **studies** across the years. F, female; M, male and U, unknown. 'NA' refers to no date record.

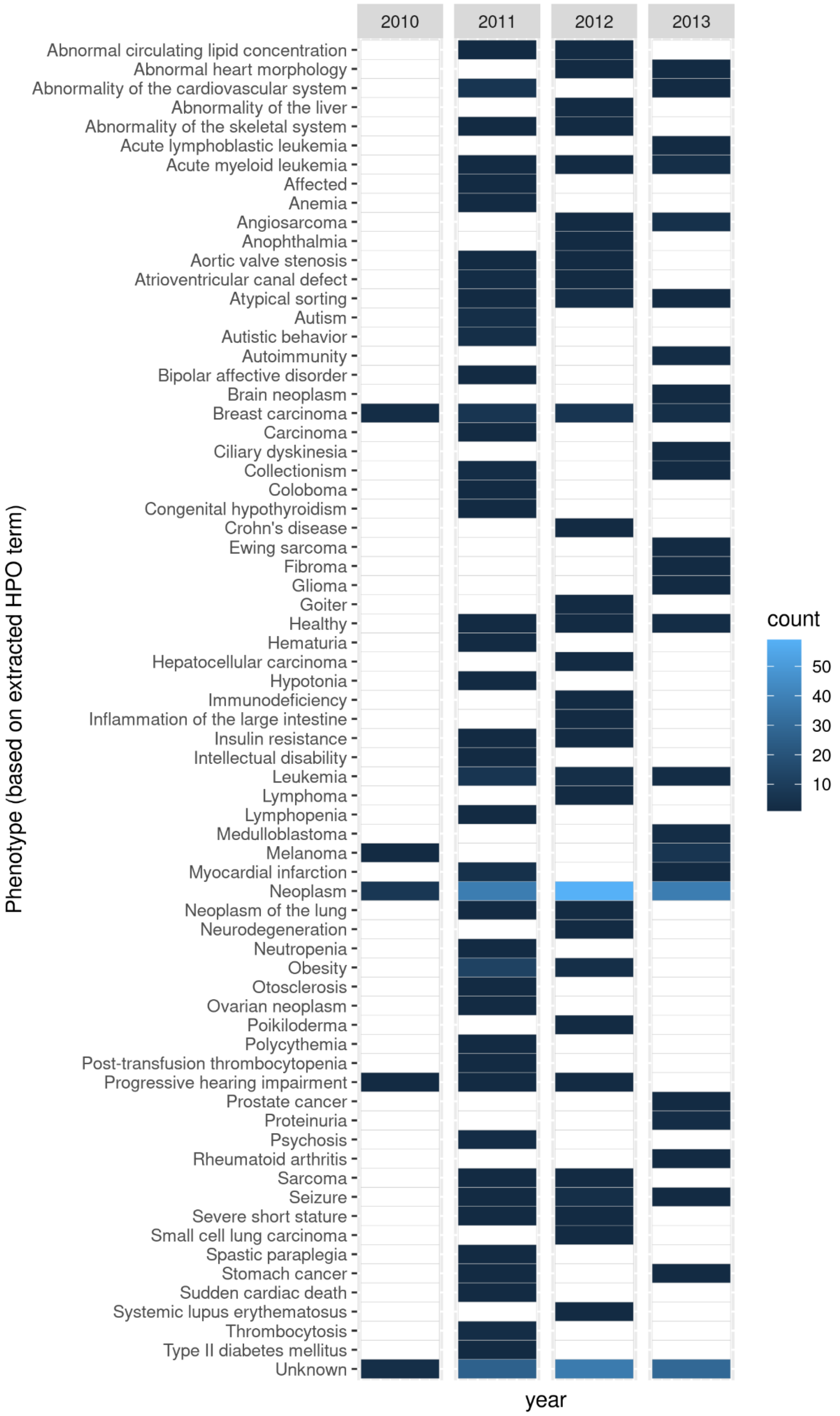

Figure S3. Phenotypes distribution in EGA studies from 2010 to 2013. Phenotypes from EGA studies (n=403) containing unknown samples only from 2010 to 2013. Detailed information on how to extract phenotypes data can be found in Methods section.

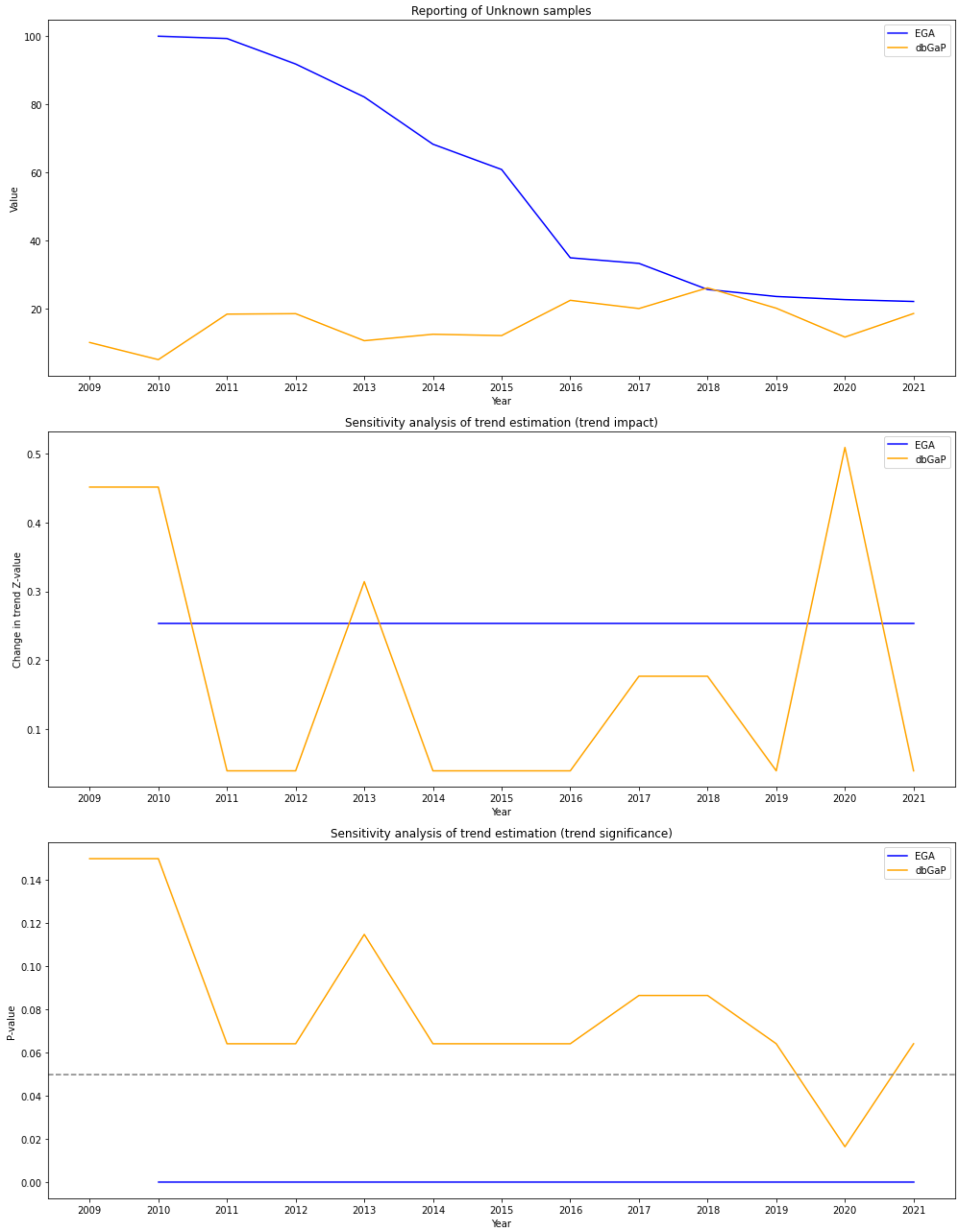

Figure S4. **Impact of potential outliers on trend analysis in yearly percentages of unknown sex samples in EGA and dbGaP**. The Modified Z-score approach did not identify any outliers. The middle panel illustrates the changes in Mann-Kendall Z-scores upon the removal of individual data points, showing consistent stability in the EGA trend and variability in dbGaP. The lower panel displays Mann-Kendall p-values upon data point removal, confirming the persistent statistical significance of the EGA trend ($p < 0.05$) and the absence of a consistent trend in dbGaP ($p > 0.05$, with exception of the removal of the year 2020, which had the highest number of samples with low unknown sex labels).

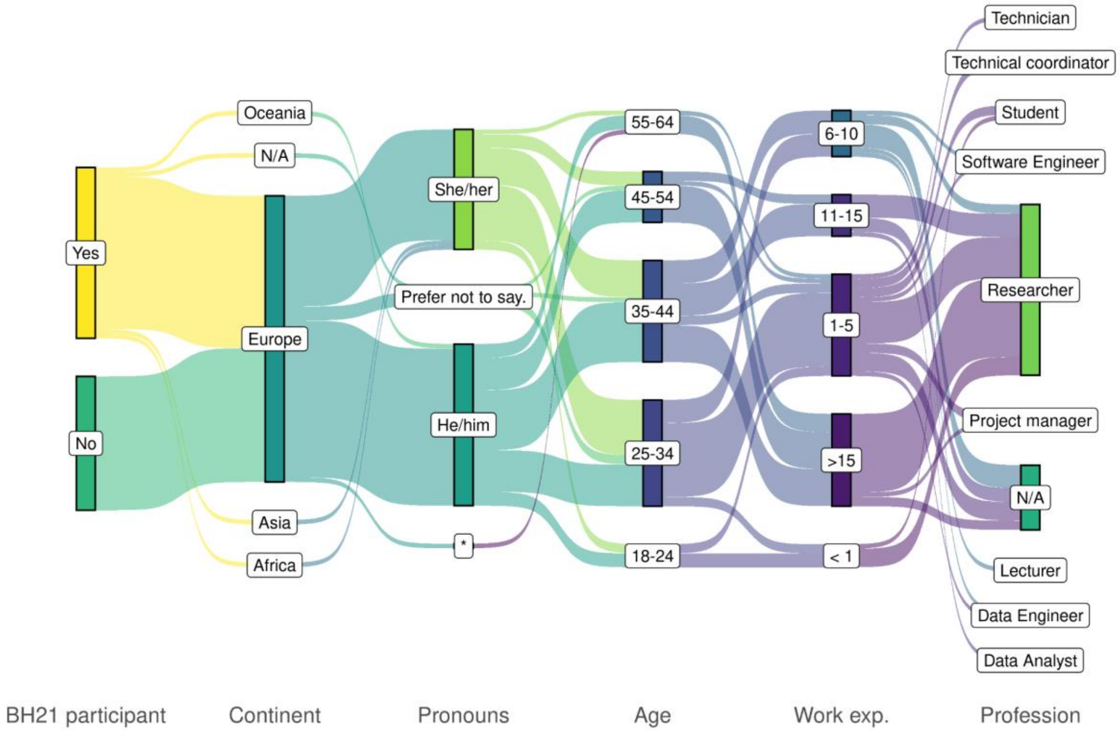

Figure S5. Sankey plot summarizing participants' socio-demographic profiles. In the pronouns column, '*' refers to "Do not subscribe to the belief system underlying this question". The "Work exp." column refers to work experience in years. We received 66 responses in total (Table S3). The majority of the respondents (n=37) participated in the Barcelona-based ELIXIR Biohackathon Europe 2021, which also had an impact on their country of origin (64% were from Spain, Figure S6). The majority of participants (n=35) identified with the pronoun "he/him", were between the ages of 25 and 44 (n=26), or had a junior profile (n=22, 1 to 5 years of experience) or a senior profile (n=20, more than 15 years of expertise). The majority of respondents (n=37) were researchers, however, their knowledge of biological databases varied. More details about the participants' profiles can be found in Table S3-S4.

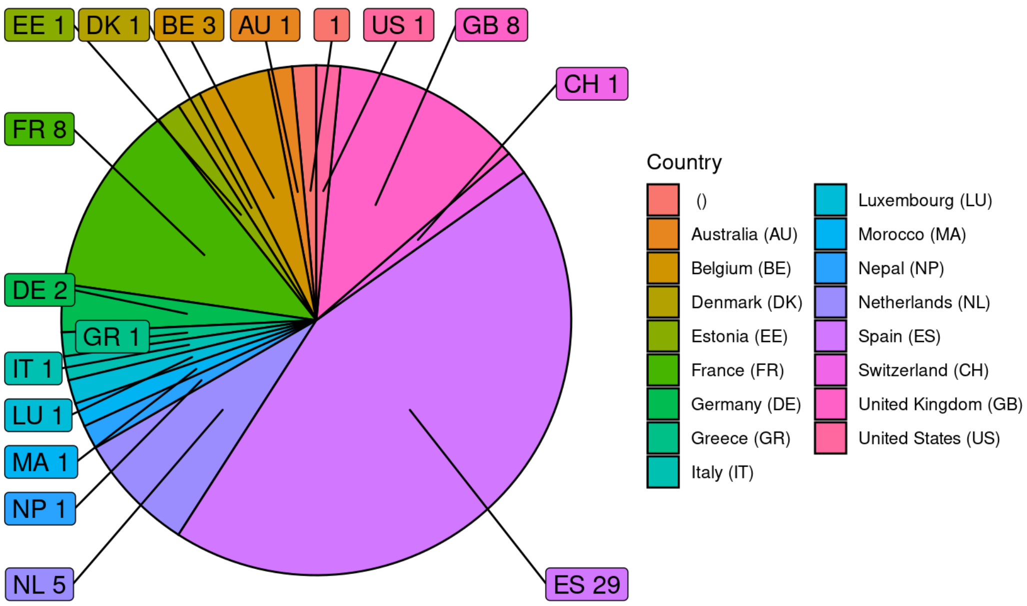

Figure S6. Questionnaire participants' place of work. Pie chart depicting participant's place of work.

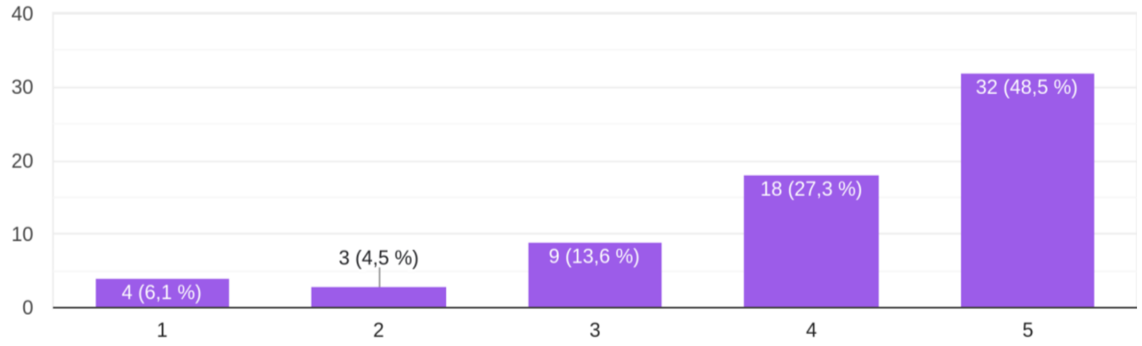

Figure S7. Summary of participants' responses to question 6. Barplot depicting survey response to question 6 ("How relevant do you consider the diversity (e.g., sex, gender, age, ethnicity) for biomedical research in your analysis?"). The numbers in the x axis goes from 1 to 5, with 1 meaning "not relevant" and 5 "very relevant". The y axis represents the number of answers.

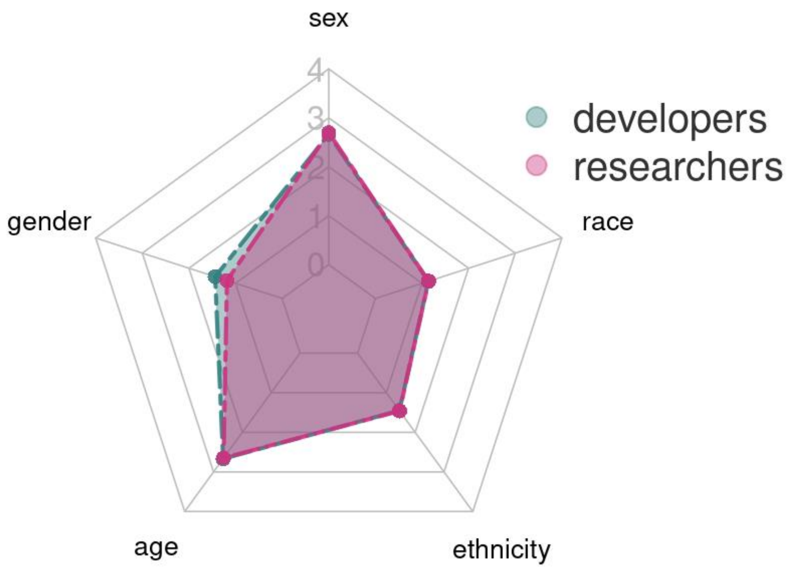

Figure S8. Summary of participants' responses to question 1 and 2. Radar chart summarizing the results of question 1 ("If you develop or maintain databases: How often these resources include the following variables in their data model?") and question 2 ("If you are a researcher: How often do you include the following variables in your datasets?"). The questions are classified by self-reported profile type, either developer or researcher. Both questions refer to variables sex, race, ethnicity, age and gender, and a numeric scale between 1 and 5 is provided for each of them to determine the frequency.

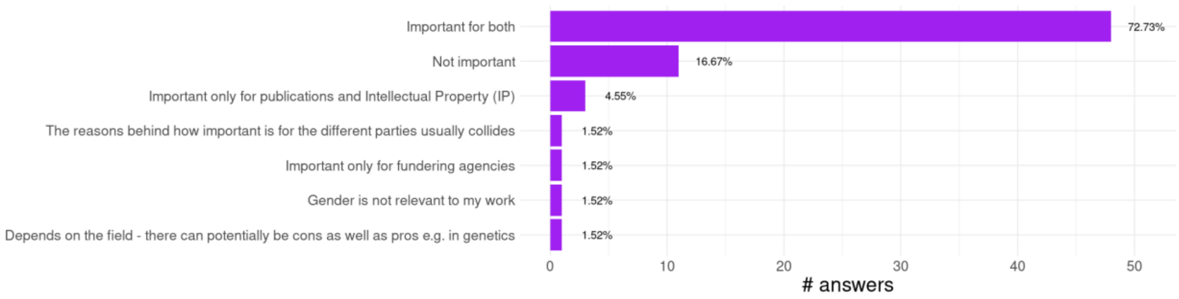

Figure S9. Summary of participants' responses to question 10. Barplot depicting survey's responses to question 10 ("How important is it to include diversity information in publication and funding processes?"). Possible answers were "Not important", "Important only for publications and Intellectual Property (IP)", "Important only for funding agencies", "Important for both", "N/A" and free text if other answer.

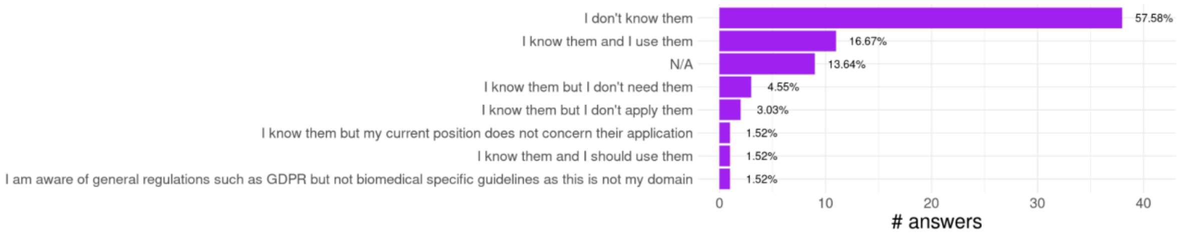

Figure S10. Summary of participants' responses to question 4. Barplot depicting participants' knowledge about diversity guidelines (question 4, “Are you familiarised with guidelines for collection sex/gender/age/race/ethnicity or recommendations of best practices in biomedical research?”).

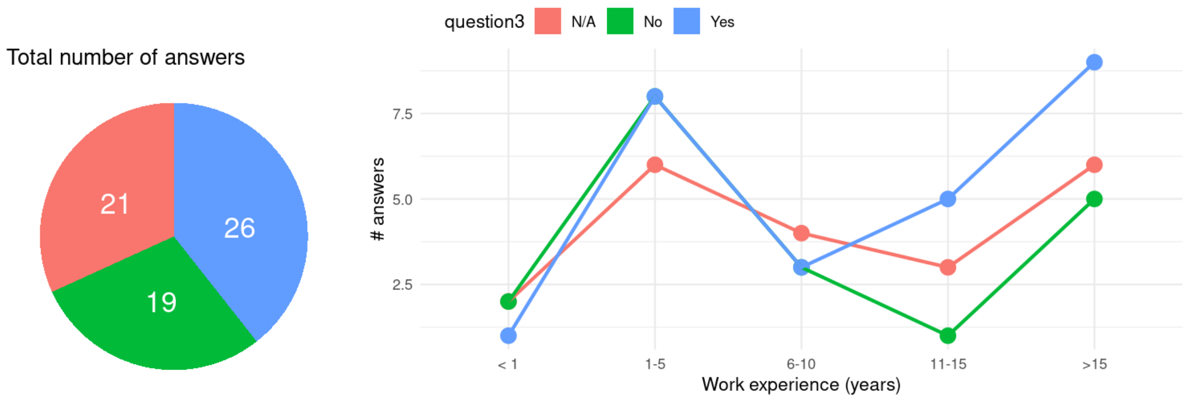

Figure S11. Summary of participants' responses to question 3. Results of question 3 ("Does your interpretation of results include a intersectional analysis (sex, gender, age, ethnicity, race)?"). The pie chart on the left represents the total number of answers per type of answer and the plot on the right depicts the same but segregated by participants' work experience.

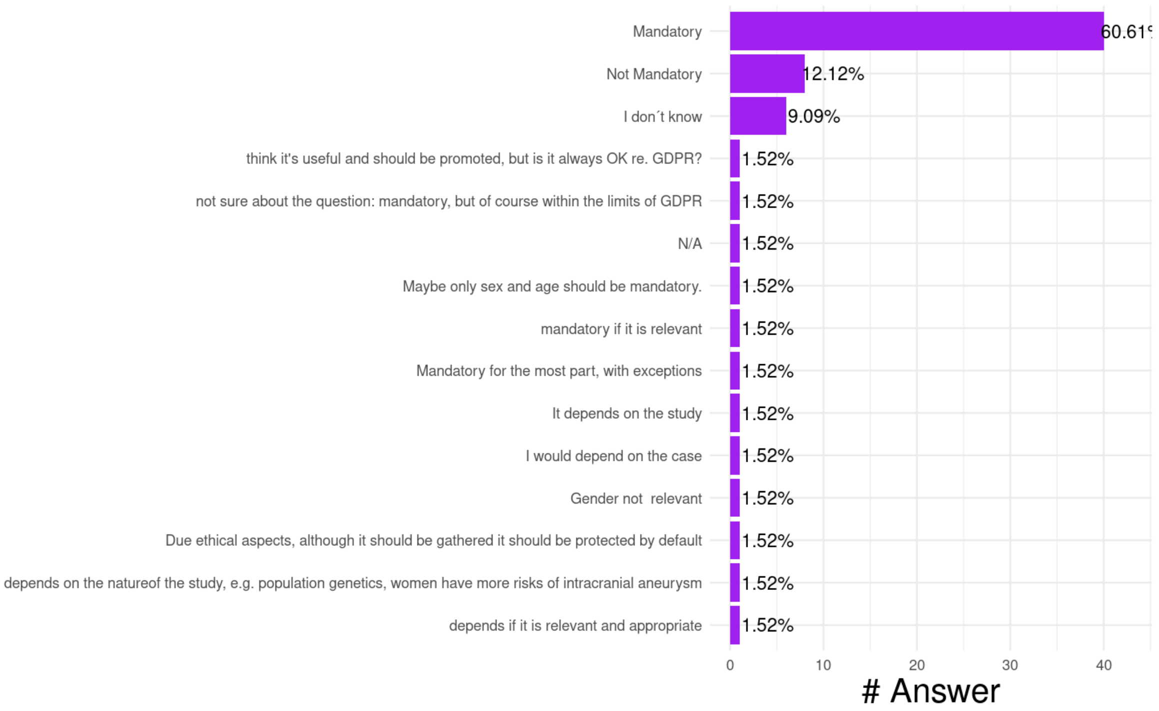

Figure S12. Summary of participants' responses to question 5. Barplot showing answers to question 5 ("Do you think capturing diversity (e.g., sex, gender, age, ethnicity) for biomedical research should be:"). While the majority of participants selected default answers provided by the questionnaire ("Mandatory", "Not mandatory" and "I don't know"), it was also possible to input free text as an answer. We received 11 individual free-text answers that can be found in Table S3.

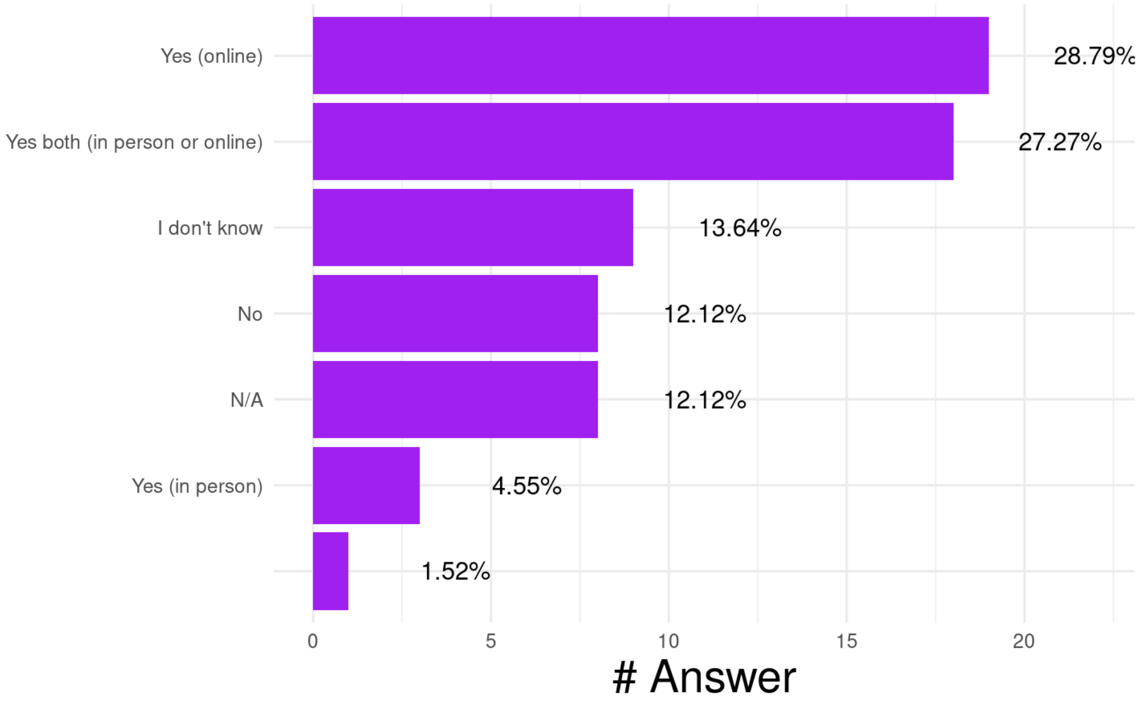

Figure S13. Summary of participants' responses to question 7. Barplot showing answers to question 7 ("Would you be interested in receiving training on social and ethical implications of biomedical research?").

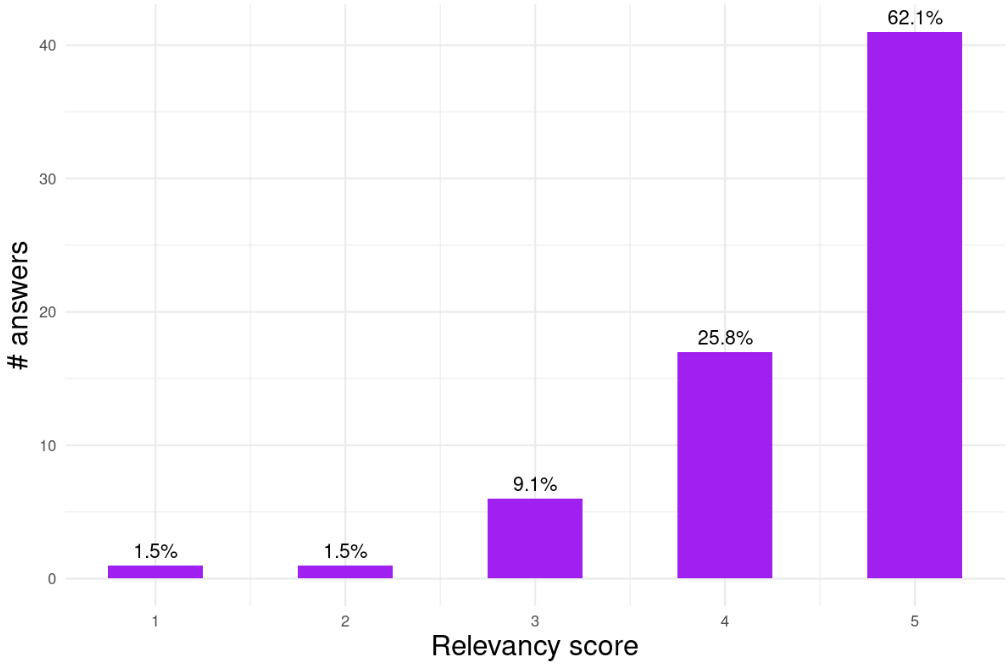

Figure S14. Summary of participants' responsesto question 9. Barplot showing distribution of answers to question 9 ("How important is educating medical data providers to help raise awareness on the importance of diversity data?"). The numbers in the x axis goes from 1 to 5, with 1 meaning "not interested" and 5 "very interested". The y axis represents the number of answers.

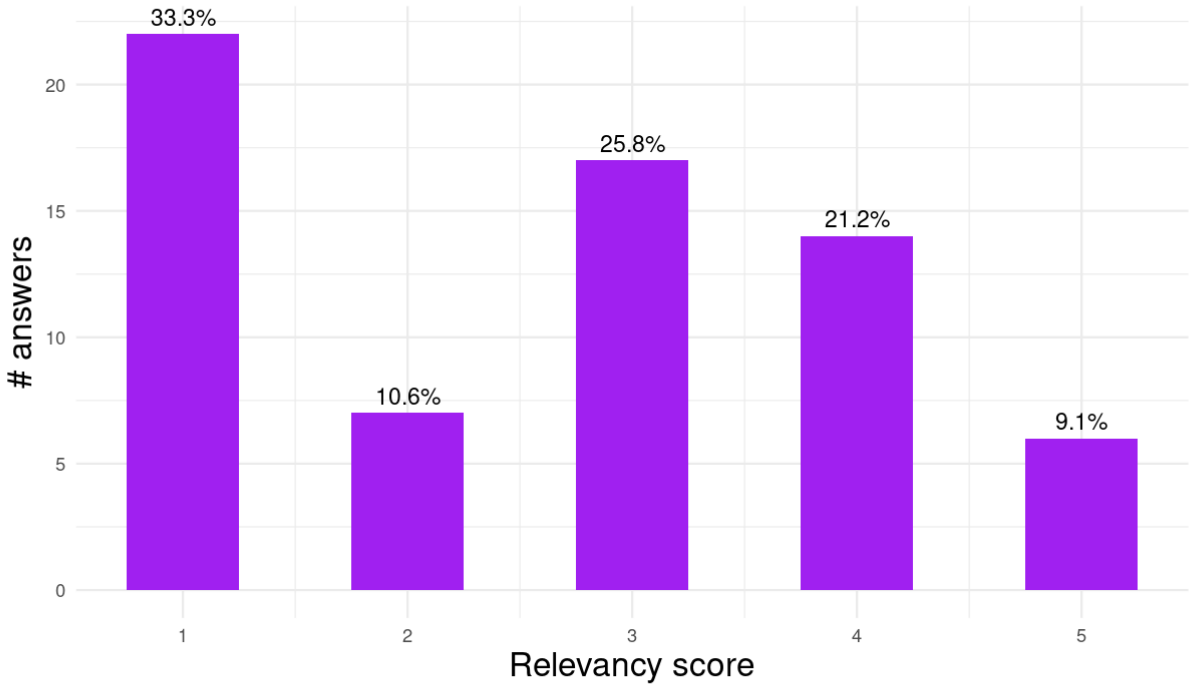

Figure S15. Summary of participants' responses to question 8. Barplot showing distribution of answers to question 8 (“Have you experienced difficulties with datasets having many missing values of diversity data due to medical data providers withdrawing (parts of) informed consent?”). The numbers in the x axis goes from 1 to 5, with 1 meaning “no difficulties” and 5 “many difficulties”. The y axis represents the number of answers.

Methods S1. Outliers analysis, related to Figure S4. Trend analysis of EGA and dbGaP unknown sex samples.

We performed a comprehensive analysis on the time-series of the yearly percentages of unknown samples in EGA and dbGaP, which are depicted as yellow lines in Figure 3B and 4B. The results of this analysis are reported in **Figure S4**.

First, we performed a **Mann-Kendall trend test**, which is a non-parametric statistical test used to identify trends in time-series. Results showed a statistically significant decrease in EGA unknowns (Mann-Kendall trend test: p-value = 8.30e-06, Z-value = -4.457) and the absence of a trend in dbGaP unknowns (Mann-Kendall trend test: p-value = 0.058, Z-value = 1.891). Then, to address yearly fluctuations in the time-series, we performed an **outlier detection analysis**. Outliers were identified using the Modified Z-score method, which is a robust anomaly detection approach to identify outliers in time-series. The Modified Z-score is calculated as follows:

$$M_i = 0.6745 \times \frac{|X_i - \mathrm{Med}(X)|}{\mathrm{MAD}}$$

where X_i represents the data point, $\mathrm{Med}(X)$ is the median of the data X, 0.6745 is a constant scaling factor representing the upper quartile of a standard normal, and MAD is the Median Absolute Deviation (MAD), defined as

$$\mathrm{MAD} = \mathrm{Med}(|X_i - \mathrm{Med}(X)|)$$

From Iglewicz and Hoaglin (1993) [S1], a data point X_i is classified as an outlier if its Modified Z-score exceeds a threshold of 3.5 (i.e. more than 3.5 times the MAD away from the median). No outliers were detected in the two time-series.

To investigate the impact of each data point on the significance of the trends, we performed a **sensitivity analysis**. For each data point X_i, the following steps were performed:

1. Remove X_i from the original time-series Y to create a modified time-series $Y' = Y \setminus \{X_i\}$.
2. Compute the Mann-Kendall Z-value for both the original Z_Y and the modified $Z_{Y'}$ time-series.

3. Determine the impact of removing X_i by calculating the absolute difference in Z-values: $|Z_Y - Z_{Y'}|$.

This process quantifies how the removal of each data point affects the statistical significance of the trend, highlighting points with an influence on the overall trend determination. The trend in EGA remains stable over the years, with an absolute change in Z-scores of 0.253 for all removed data points, indicating consistent robustness (Figure S4, middle panel). In contrast, the trend in dbGaP shows variability, underscoring the absence of a consistent trend in this dataset (Figure S4, middle panel). Furthermore, the removal of individual data points does not affect the statistical significance of the trends for either dataset; the p-values remain below 0.05 for EGA and above 0.05 for dbGaP with the exception of the year 2020 (Figure S4, lower panel), which had the highest number of samples with low unknown sex labels (see Figure 3A). Removing the year 2020 in the dbGaP data confers statistical significance to the estimation of a slightly positive trend in unknown sex labels (Mann-Kendall trend test: p-value = 0.016, Z-value = 2.4), confirming how important is to introduce actions to systematically reduce unreported cases.

Methods S2. Qualitative analysis, related to Figures S5-S15. Supplemental qualitative analysis at ELIXIR BioHackathon Europe 2021 mentioned in Results section.

The primary goal of this analysis was to encourage a proper systematic reporting of sex and gender categories in human data repositories for biomedical research. In this sense, users and database developers are responsible for making sex and gender aspects visible by adopting good research practices, including the integration of diversity (i.e. representation of a wide range of characteristics including sex, gender, age, race, ethnicity, etc.). Thus, we performed a qualitative analysis (see Methods) to assess people's knowledge of sex and gender bias in data repositories and the implications of these biases, an anonymous survey was distributed.

We received 66 responses in total (Table S3A). The responses were reformatted for plotting purposes (Table S3B). Figure S4 depicts a summary of the survey participants' characteristics. The majority of the respondents (n=37) participated in the Barcelona-based ELIXIR Biohackathon Europe 2021, which also had an impact on their country of origin (64% were from Spain, Figure S6). The majority of participants (n=35) identified with the pronoun "he/him", were between the ages of 25 and 44 (n=26), or had a junior profile (n=22, 1 to 5 years of experience) or a senior profile (n=20, more than 15 years of expertise). The majority of respondents (n=37) were researchers, however, their knowledge of biological databases varied. More details about the participants' profiles can be found in Table S3B.

Among the results, it stands out that the majority (n=50) apply intersectional analysis in their work, considering diversity in research as extremely relevant (n=32) or very relevant (n=18) (question 6, Figure S7). Both maintainers and researchers are more prone to consider sex and age in their data than race, gender and ethnicity variables (questions 1 and 2, Figure S8). It should be noted that the survey did not make any distinction between animal and human samples. It is controversial that although nearly 70% of the respondents considered it important to include diversity in scientific publications as well as in financing processes (question 10, Figure S9), 67% of all participants were unaware of the ethical guidelines or international reference manuals regarding the inclusion of sex and gender diversity in science (question 4, Figure S10). Lastly, we found that participants with more work experience tended to include more intersectional analysis (sex, gender, age, ethnicity, race) in their studies compared to those with less experience (question 3, Figure S11). However, this finding does not affect the common belief (61% of participants) that the variable sex and gender should be mandatory in biomedical research (question 5, Figure S12).

In summary, there is a need to continue providing training, resources on ethical tools, and guidelines for inclusion of sex and gender in science (question 7 and 9, Figure S13 and S14 respectively) as it was noted that retrieval of data is not always as complete as it should be (question 8, Figure S15). Additionally, the use of the variables such as race or ethnicity should become mandatory to have more inclusive and effective results in health research. It is key to continue with this type of exploration study in the academic area and companies in healthcare fields, to focus on new strategies of dissemination and training for people who use genomic data repositories.

Despite the limitations of the results from the survey, these insights provide valuable context for understanding the complexities of research bias and underscore the importance of ensuring adequate representation in scientific studies.

Supplemental References

[S1] Iglewicz, B., and Hoaglin, D.C. (1993). How to detect and handle outliers (ASQC Quality Press).
